# Transmission electron microscopy characterisation of Spirulina bioplastics

**DOI:** 10.1111/jmi.70093

**Published:** 2026-04-15

**Authors:** Sourena Azidhak, Douglas J. Taatjes, Ian R. Campbell, Eleftheria Roumeli, Linda S. Schadler

**Affiliations:** ^1^ Department of Mechanical Engineering University of Vermont Burlington Vermont USA; ^2^ Department of Pathology and Laboratory Medicine Microscopy Imaging Center University of Vermont Burlington Vermont USA; ^3^ Department of Material Science and Engineering University of Washington Seattle Washington USA

**Keywords:** biomaterial, bioplastic, cyanobacteria, microalgae, Spirulina, TEM

## Abstract

Plastic pollution is impacting global ecosystem health. Bioplastics made from unprocessed biomass, including whole or fragmented tissues, are one potential solution. They are biodegradable and exhibit competitive mechanical performance. Seaweed and microalgae have gained popularity over the last decade as bioplastic feedstocks due to their abundance and ease of cultivation. To grasp the complexity of transforming biomatter into bioplastics and to optimise their performance, it is essential to develop processing–structure–property relationships. Transmission electron microscopy can be used to observe the morphology of bioplastics; however, current infiltration techniques are designed for biomaterials in their natural state, and harsh reagents can cause artefacts in bioplastic structure. This paper examines four different fixation methods for Spirulina bioplastics and investigates the impact of fixative, contrasting, dehydration, and resin embedding on Spirulina ultrastructure before and after bioplastic transformation. Our study suggests that the key to preserving bioplastic morphology is to prepare the specimen for electron microscopy by exposing it to gas‐phase infiltration.

## 1 INTRODUCTION

Bio‐based plastics are an emerging class of materials that can serve as suitable substitutes for petroleum‐based plastics. Generally, bioplastics are biodegradable, do not persist in the environment as microplastics, and exhibit mechanical characteristics similar to synthetic plastics.^1^, ^2^, ^3^, ^4^ Biomass from various organisms, including plants, animals, and microbes, can be transformed into bioplastics either by extracting specific biopolymers (extraction method) or by processing whole tissues and cells (direct method).^4^, ^5^ For instance, protein isolated from Spirulina cyanobacteria can be cast to form a degradable biofilm^6^ or its biomass can be converted directly into a bioplastic through thermal processing without additional steps.^3^ Fabricating bioplastics directly from whole biomass can save time and energy associated with extraction and can limit waste production.^4^

**FIGURE 1:**
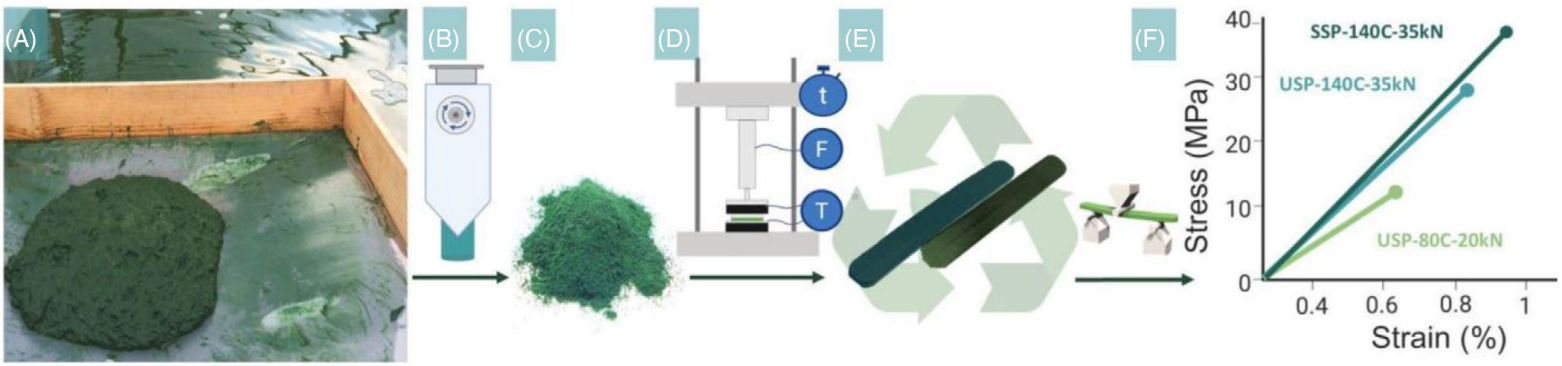
The transformation of Spirulina from native biomass into a processed bioplastic is illustrated as follows: (A) Spirulina cyanobacteria grow suspended in the water; after a dehydration step (B), dried Spirulina powder (C) is obtained. The powder undergoes thermomechanical processing at specific time (*t*), force (*F*) and temperature (*T*) via hot pressing (D), yielding a biodegradable, recyclable bioplastic (E) whose mechanical properties depend on processing conditions (F). Flexural testing results are shown for bioplastics produced from unprocessed Spirulina (USP) powder, hot‐pressed at 140°C‐35 kN and 80°C‐20 kN, as well as from sonication‐induced Spirulina (SSP) powder (image (C) was captured in our lab. Spirulina pool image (A) was adopted from smartmicrofarms.com. Mechanical property data (F) are obtained from Iyer et al.^3^, ^20^).

The cyanobacterium *Limnospira platensis* (formerly known as *Arthrospira platensis* ^7^, ^8^, ^9^, ^10^, ^11^) is of special interest as a feedstock for mass production of bioplastics, as it grows rapidly and does not compete with agricultural products for arable land.^1^, ^3^, ^4^ *Limnospira platensis* is colloquially and commercially referred to as ‘Spirulina’, and it will be described as such in this article. Spirulina is a filamentous, blue‐green, prokaryotic cyanobacterium (that is also often considered a microalga in the literature^3^, ^12^, ^13^) commonly found in alkaline or natural waters across regions of China, Africa, and Mexico.^9^, ^14^, ^15^ It is composed of various biopolymers, including 50%–70% protein, 5%–10% lipid, and 15%–20% carbohydrates by dry weight, as well as trace amounts of vitamins, minerals, pigments, polyhydroxyalkanoates (PHA), and secondary metabolites such as phenolic acids and carotenoids.^3^, ^14^, ^16^, ^17^ Recent work has demonstrated that applying elevated temperature (*T*) and force (*kN*) for short durations (*t*) (Figure 1D) to dried Spirulina powder (Figure 1C) results in a direct transformation of the cellular biomass into a cohesive bioplastic (Figure 1E).^3^, ^12^, ^18^ Spirulina bioplastics are recyclable, backyard‐compostable, and demonstrate strength and stiffness (𝜎 = 26 MPa, *E* = 2.6 GPa) (Figure 1F) comparable to conventional plastics like polyethylene (PE: 𝜎 = 10–32 MPa, *E* = 0.25–1.25 GPa) and polypropylene (PP: 𝜎 ≈ 26 MPa, *E* ≈ 2 GPa).^3^, ^19^

Despite their advantageous properties, the relationship between microstructure and performance of Spirulina bioplastics has not been rigorously characterised. Understanding the connection between processing and microstructure is essential for optimising the mechanical properties of these materials and is therefore of special interest. This involves characterising the microstructure, bonding, cell‐component interactions, and overall biomass‐to‐bioplastic ultrastructural transformation, as well as observing mechanical failure mechanisms.

Typically, scanning electron microscopy (SEM), Fourier transform infrared spectroscopy, and bulk mechanical testing have been used in conjunction to assess the impact of processing conditions, microstructure, and chemical interactions on the performance of Spirulina bioplastics.^3^ However, these techniques do not provide direct evaluation of bioplastic ultrastructure morphology or cell‐level interactions. Transmission electron microscopy (TEM), when performed correctly, would enable direct identification of thermomechanical processing effects on the transformation of biomass to bioplastic.^4^

To obtain TEM images with fidelity, it is essential to use a sample preparation procedure that preserves the specimen's morphology/ultrastructure and protects it from electron beam damage.^21^, ^22^ General histochemistry and cytochemistry methods for preparing plants, microalgae, animal tissues, and cells for TEM include fixation, contrasting, dehydration, and resin embedding.^23^, ^24^, ^25^ Each of these preparative (infiltration) steps involves applying various chemicals to organic specimens, and it is important to understand the interactions between each chemical and the Spirulina bioplastics to ensure structural fidelity during TEM.

The primary fixative is the initial histochemical step that maintains cell ultrastructure and structural integrity by chemical bonding to the tissue.^22^, ^26^ Aldehydes, such as formaldehyde (FA) and glutaraldehyde (GA), are cross‐linking fixatives that preserve cellular structures by forming methylene bridges between proteins and nucleic acids.^13^, ^26^, ^27^ In addition to covalent reinforcement, aldehydes can also extract certain biopolymers, including acidic proteins and phospholipids, during fixation.^28^, ^29^ The generation of hydrogen ions during aldehyde‐amine interactions lowers the solution pH and can further enhance biopolymer extraction by weakening non‐covalent interactions.^28^, ^30^, ^31^ Sodium cacodylate buffer is commonly used in aldehyde mixtures to limit the pH‐driven extraction during fixation.^31^, ^32^

Post‐fixation, or contrasting, alters the electron density and stabilises particular components such as lipid‐containing cellular structures.^22^, ^31^, ^33^ Osmium tetroxide (OsO_4_) is a water‐soluble heavy‐metal (electron‐dense) complex that can serve as both a contrasting agent and fixative by inducing inter‐ and intramolecular crosslinking between specific biomolecular components.^22^, ^34^ It usually reacts with the unsaturated bonds of phospholipids and lipids, hydrophobic and hydrophilic sites on protein side chains, and/or cross‐links between proteins and lipids.^22^, ^34^, ^35^

To transfer the specimen from a hydrophilic medium to a hydrophobic resin, such as Spurr's resin, organic samples must undergo dehydration by applying coagulant fixatives, such as ethanol, acetone, and propylene oxide. Dehydrants such as ethanol and methanol remove free water from the specimen, destabilising proteins’ hydrophobic interactions and hydrogen bonds, disrupting protein tertiary structure, and leading to partial protein denaturation.^22^, ^26^, ^28^ While ethanol has a higher infiltration rate than other dehydrants like acetone, it can dissolve lipids and cause sample shrinkage.^36^, ^37^

Because of these unfavourable interactions between alcohol dehydrants and lipids and proteins, acetone is the dehydrating agent of choice for immunohistochemistry and enzymes. However, acetone can also cause sample shrinkage, cracking, and hardening. Propylene oxide is an alternative dehydrant that has better miscibility with Spurr's resin than acetone. Improved miscibility with the resin facilitates the resin's infiltration into the sample, which is beneficial for dehydrating larger and denser tissues.^30^, ^36^, ^37^, ^38^

After gradual dehydration, organic specimens are typically transferred slowly to an epoxy or acrylic resin. Epoxy resins, like Spurr's resin, have low viscosity and relatively rapid penetration, providing high electron‐beam resistance and facilitating sample sectioning.^39^ Acrylic resins, such as Lowicryl, are less viscous than epoxy resins, allowing for faster penetration. Despite their advantages, some acrylic resins, such as Lowicryl K4M and HM20, are water‐miscible and hydrophilic, which creates challenges while sectioning in water.^24^, ^40^ UV light polymerisation is a popular protocol for embedding biological specimens in acrylic resins for post‐embedding immunoelectron microscopy.^41^ However, using a contrasting agent or darker samples is unsuitable for this method; an alternative is heat‐induced polymerisation.^42^

Biological samples are often further exposed to chemical reagents during post‐contrasting (double contrasting). Ultrathin sections are treated with uranyl acetate followed by lead citrate to improve contrast. Uranyl acetate primarily interacts with lipids, proteins, and nucleic acids through binding to phosphate, amino, and carboxyl groups. Lead citrate improves contrast further by binding to negatively charged molecular groups and structures previously treated with OsO_4_. Lead citrate also interacts weakly with uranyl acetate; therefore, it is conventionally applied after uranyl acetate, contrasting.^37^, ^43^

Taken together, these sample preparation protocols developed to study various types of algae, microalgae, and plants feature the application of numerous harsh chemicals.^21^, ^44^, ^45^, ^46^ While these methods were specifically designed to preserve the native morphology of biological cells and tissues, imaging previously performed on compressed plant cells suggests that once biological systems undergo thermomechanical processing to form bioplastics, these chemical protocols can induce void formation and significant morphological distortion.^4^, ^47^ Such artefacts compromise the TEM image fidelity and hinder the establishment of reliable processing–structure–property relationships.

In this study, we address a critical methodological gap through the comparative analysis of TEM fixation protocols and the resulting fixation‐induced artefacts. We develop a robust method that enables high‐fidelity imaging of processed (transformed) bioplastics independent of the selection of processing conditions. The objective is to minimise infiltration‐induced artefacts, such as holes and resin penetration, within the matrix to reveal a faithful representation of bioplastic morphology. This approach enables direct observation of cell disassociation, cell–cell interactions, and thermomechanical‐processing‐induced changes in cellular components relative to the native state, as demonstrated here using a Spirulina‐based bioplastic.

## 2 MATERIALS AND METHODS

### 2.1 Bioplastic preparation

The Spirulina bioplastic production process is depicted in Figure 1. The material feedstock for this study was organic Spirulina (Nuts.com). According to the product description, the cyanobacteria are cultivated in shallow and open‐channel raceway ponds in China (Figure 1A). Once the Spirulina culture reaches the appropriate density, it is harvested using fine filtration, rinsed with clean water, and dried by spray drying (Figure 1B) to obtain the final Spirulina biomass in a powder form (Figure 1C).^48^

Prior to bioplastic fabrication, as‐received, unprocessed Spirulina powder (USP powder) (shown in Figure 1C) was allowed to equilibrate at 40% relative humidity and room temperature. Afterward, the powder was compression‐moulded in a 4122 Carver hot‐press (Figure 1D) using a specific time (*t*), temperature (*T*), and force (*kN*). Custom stainless‐steel moulds were loaded with 1 g of Spirulina and subjected to elevated heat and pressure to produce bioplastic samples approximately 60 mm long, 8 mm wide, and 2 mm thick (Figure 1E).

In this study, two types of Spirulina bioplastics were analysed. USP‐140°C‐15 kN specimens were made via hot‐pressing USP powder at 140°C and 15 kN for 5 min, where USP powder contains a mixture of intact and fragmented cells, trichomes and impurities. Additionally, BMSP‐140°C‐7 kN bioplastic samples were made via hot pressing ball‐milled Spirulina powder (BMSP powder) at 140°C and 7 kN for 5 min. BMSP powder comprises fully disrupted Spirulina cells and trichomes. The BMSP‐140°C‐7 kN bioplastic sample was added to observe the impact of cell disruption on the resulting Spirulina bioplastic structure and to ensure that the TEM methods developed here are robust enough to correctly image different Spirulina bioplastic microstructures.

To prepare cell‐disrupted BMSP powder, USP powder underwent additional preprocessing prior to humidity incubation. A container of Spirulina was submerged in liquid nitrogen (LN_2_) for 5 min to embrittle the powder. Then, the dried powder was added to a stainless‐steel jar with stainless steel media (MSE Supplies) and was milled at 600 rpm for 1 h using the MSE PRO 1L High Energy Ball Mill. Following a 20‐min rest (to avoid overheating), the powder was milled for another 1 h at 600 rpm.

### 2.2 Scanning electron microscopy

Spirulina bioplastics were fixed to aluminium stubs using carbon tape (Ted Pella). To ensure conductivity, the surface of each bioplastic sample was sputter‐coated with 4 nm gold using the 108 Manual sputter coater from Ted Pella. Sputtered Spirulina bioplastics were imaged with the Phenom ProX Desktop SEM (Thermo Fisher Scientific) using an accelerating voltage of 10 kV.

### 2.3 Bright‐field microscopy

As‐received Spirulina powder was wet‐mounted in deionised (DI) water between a microscope slide and a cover slip. Mounted slides were imaged on a Panthera TEC‐BF (Motic) using a 5× objective.

### 2.4 TEM sample preparation protocols

Prior to sample fixation, OsO_4_ solution, lead citrate, 0.1 M cacodylate solution, Spurr's resin, and Lowicryl resin were prepared. The OsO4 solution was prepared by diluting 4% osmium crystals (Electron Microscopy Sciences [EMS]) in distilled water overnight. Lead citrate solution was prepared by dissolving 1.33 g lead (II) nitrate (EMS) and 1.76 g sodium citrate (EMS) in 30 mL DI water, followed by thorough mixing and incubation for 30 min. Eight millilitres of 1 M NaOH (EMS) were then added, and the solution was brought to a final volume of 50 mL with DI water. The mixture was ultrasonicated until all salts were fully dissolved and stored at room temperature until use.^49^

A 0.1 M cacodylate solution (pH 7.2) was prepared by combining sodium cacodylate and hydrogen chloride (EMS). Spurr's resin was prepared by mixing 10 g ERL‐4221 (cycloaliphatic epoxide resin [EMS]), 10 g DER‐736 epoxy resin (EMS), 26 g nonenyl succinic anhydride (EMS), and 0.4 g 2‐dimethylaminoethanol (EMS) as a hardener. The mixture of the components yielded approximately 45 mL of resin, which could be stored at −20°C in sealed tubes prior to use. Unused resin and contaminated waste were polymerised at 70°C overnight before disposal.^50^

Lowicryl K4M resin was prepared using an EMS kit. Crosslinker A (2.7 g) and Monomer B (17.3 g) from the Lowicryl K4M kit were mixed gently. Then, a dry nitrogen gas stream was used to mix the resin at a low rate for several minutes. The N_2_ gas also prevents the incorporation of oxygen, which interferes with the resin polymerisation process. Next, 0.3% Dibenzoyl peroxide (DBPO) (Fisher Scientific) by weight was added to the mixture (instead of Initiator C) and stirred under N2 until no DBPO residue was visible.^42^ These components were utilised during TEM sample preparation.

Table 1 shows the TEM preparation methods (A–E) and the corresponding microtomy thicknesses for resin‐embedded Spirulina powder and bioplastics. Method A was used to prepare dry USP powder. Methods B–E were used to evaluate the impact of various protocols on the morphology and ultrastructure of Spirulina bioplastics (USP‐140°C‐15 kN). To examine the applicability of Method E beyond bioplastics made from USP powder, Method E1 was also used to prepare a cell‐disrupted bioplastic sample (BMSP‐140°C‐7 kN). Below, each sample preparation method is described in detail.

**TABLE 1:** Overview of microtomy thicknesses and TEM preparation protocols used for dried Spirulina (USP powder) (Method A), a USP bioplastic, USP‐140°C‐15 kN (Methods B–E), and a cell‐disrupted bioplastic specimen, BMSP‐140°C–7 kN (Method E1). Detailed descriptions of all techniques are provided in the methodology section.

	Fixation (aldehyde and contrasting)		Dehydration	Embedding resin	Double contrasting	Microtomy thickness (nm)
**A**	3% glutaraldehyde (2 h)	2% OsO_4_ (1 h)	Ethanol propylene oxide	Spurr's	uranyl acetate lead nitrate	A: 70–85
**B**	3% glutaraldehyde (2 h)	2% OsO_4_ (1 h)	Ethanol propylene oxide	Spurr's	–	B‐C: 80–100
**C1**	2% OsO_4_ (1 h)		Ethanol acetone	Spurr's	–	
**C2**	2% OsO_4_ (1 h)		Ethanol propylene oxide	Spurr's	–	
**D**	2% OsO_4_ vapour (24 h)		–	K4M Lowicryl	–	D: 140–170
**E1**	2% OsO_4_ vapour (24 h)		–	Spurr's	–	E: 100–130
**E2**	2% OsO_4_ vapour (48 h)		–	Spurr's	–	

Method A was adapted from studies of fresh Spirulina cyanobacteria and was applied here to characterise the native structure of dried Spirulina powder (USP powder).^11^, ^14^, ^51^ A few milligrams of the powder were placed onto low‐melting agarose (3% wt agarose powder dissolved in distilled water) (SeaPrep Agarose) on a dental wax sheet(EMS). After thorough mixing, the sample was stored at 4°C for 15 min. To solidify the agarose faster, the gel was fixed in 3% glutaraldehyde (EMS) in 0.1 M cacodylate buffer and stored at 4°C for 15 min.^52^

The embedded Spirulina powder in agarose gel was then sectioned into 0.5–1 mm^3^ pieces and kept in 3% glutaraldehyde in 0.1 M cacodylate buffer for 2 h, followed by rinsing three times with the same buffer for 10 min to remove the fixative remnants. Next, the samples were fixed for 1 h in a 2% OsO_4_ (EMS) solution diluted from a 4% stock with 0.1 M cacodylate buffer, followed by rinses in the same buffer (3 × 10 min each) to remove any contrasting reagent residue. The samples were then dehydrated in a graded series of ethanol solutions (ThermoFisher Scientific) (50%, 70%, 85%, and 100%) for 5 min each and rinsed with 100% propylene oxide (ThermoFisher Scientific) for 10 min (2 times).

Samples were sequentially transferred to 3:1 and 1:1 propylene oxide:Spurr's resin solutions and kept in a tube rotator for 1 h during each step. Next, specimens were moved into 1:3 propylene oxide:Spurr's resin and left to rotate overnight. The next day, samples were rotated in pure resin for 1 h, then embedded in a silicon mould and polymerised at 70°C overnight. After the microtomy step described in section 2.5, the thin section was contrasted with 2% uranyl acetate (EMS) in 50% ethanol solution, followed by lead nitrate.

Method B was used for thermomechanically processed Spirulina bioplastic (USP‐140°C‐15 kN). None of the methods for bioplastic (B to E) included the agarose embedding step, as the material was sufficiently thick (0.2–0.5 mm^3^) to be preserved during infiltration. The infiltration sequence in Method B was the same as in Method A, with the exception of the double contrasting step. In fact, this step could not be implemented during Methods B–E as the bioplastic samples detached from the TEM grid and were washed away due to the fragile nature of thin sections.

The focus of Methods C1 and C2 was to assess the impact of contrasting and different dehydrating agents on bioplastics (USP‐140°C‐15 kN) without the aldehyde fixative step. Method C1 resembles a method reported for observing the morphology of processed tobacco cell biocomposites, except with a longer acetone dehydration time.^47^ For C1 and C2, small bioplastic samples (0.2–0.5 mm^3^) were kept in 2% OsO_4_ solution with 0.1 M cacodylate buffer for 1 h and then rinsed 3 times in buffer for 10 min. After contrasting, samples prepared using Method C1 were dehydrated in a graded series of ethanol (50%, 70%, 90%, and 100%) for 5 min each, then placed in 100% acetone (ThermoFisher Scientific) twice for 10 min each. Next, the samples were sequentially transferred into 3:1, 1:1, and 1:3 acetone:Spurr's resin mixtures and rotated on a tube rotator for 1 h at each step. Finally, the samples were rotated in 100% Spurr's resin overnight, embedded in a silicon mould, and polymerised at 70°C overnight.

For Method C2, after the osmication, rinsing, and ethanol dehydration steps (same as C1), samples were rinsed twice for 10 min with 100% propylene oxide. Next, samples were sequentially transferred to 3:1, 1:1, and 1:3 propylene oxide:Spurr's resin solutions and rotated for 1 h at each step. Finally, the samples were rotated in 100% Spurr's resin overnight, and the next day, they were embedded in fresh resin and polymerised at 70°C overnight.

In Method D, the lower viscosity of Lowicryl K4M resin compared to Spurr's resin motivated us to explore the heat‐induced Lowicryl polymerisation method on Spirulina bioplastic (USP‐140°C‐15 kN).^40^, ^42^ In addition to using a different embedding resin, we also eliminated the dehydrating step for this and the following procedure. To contrast the material, the samples were exposed to 2% OsO4 water vapour for 24 h at room temperature. The vapour fixation technique was developed for delicate tissues, such as neurons or lipid‐containing membranes, to avoid direct interaction with harsh liquid chemicals.^33^, ^39^, ^40^

For vapour fixation, bioplastic pieces (0.5–1 mm^3^) were held with tweezers and secured with tape to prevent sample loss, then suspended above a small glass vial containing a few millilitres of 2% OsO_4_ diluted in distilled water without contacting the solution. The vial was sealed with Parafilm and maintained at room temperature for 24 h to allow vapour‐phase fixation. If OsO_4_ droplets formed on the sample during vapour exposure, they were rinsed away with water. Next, the osmicated samples were broken down into smaller pieces, transferred to the Lowicyl K4M solution, and placed on a tube rotator overnight. The next day, the samples were embedded in a gelatine capsule filled with K4M to reduce oxygen penetration and recapped. Samples were then held at 60°C for 2–3 days to ensure Lowicryl polymerisation.^42^

For Methods E1 and E2, the effects of varying OsO_4_ vapour fixation time were studied. For Method E1, bioplastic samples (USP‐140°C‐15 kN) were contrasted with 2% OsO_4_ vapour for 24 h using the vapour fixation protocol described earlier. The next day samples were broken down into smaller pieces, transferred to pure Spurr's resin, rotated in a tube rotator overnight to enhance infiltration, and then embedded in Spurr's resin at 70°C overnight. In addition to USP bioplastic specimen, a cell‐disrupted Spirulina bioplastic (BMSP‐140°C‐7 kN) was prepared and contrasted with 2% OsO4 vapour for 24 h. We refer to this specimen as ‘BMSP‐140°C‐7kN’ throughout this paper. For E2, the same vapour fixation procedure was applied except that the samples (USP‐140°C‐15 kN) were exposed to 2% OsO_4_ vapour for 48 h and then embedded in Spurr's resin with the same steps described for Method E1.

### 2.5 Sectioning

Table 1 summarises the microtomy thickness utilised for each sample to achieve a gold‐to‐silver interference colour suitable for TEM imaging. After resin embedding, all specimens were trimmed and sectioned using a Reichert Ultracut ultramicrotome. The embedded resin surface was first smoothed with a razor blade, then fine‐trimmed with a glass knife. For the USP powder (Method A), ultrathin sections, approximately 70–85 nm thick, were successfully obtained using a DiATOME Ultra 45° diamond knife.

Methods B–E were applied to Spirulina bioplastic samples. The Ultra diamond knife frequently produced sections that disintegrated in water, reflecting the fragility of the fixed bioplastic matrix. To mitigate this, a DiATOME Histo diamond knife was used instead. This blade generated slightly thicker, but more stable, sections necessary to preserve microstructural continuity during cutting. Spurr's resin–embedded specimens prepared using Methods B‐C were sectioned at 80–100 nm.

For the Lowicryl K4M–embedded specimens (Method D), sectioning required a reduced water level in the diamond knife boat, as excess water softened the highly hydrophilic Lowicryl matrix and disrupted section integrity. As such, sections were cut at 140–170 nm. While this thickness is thicker than conventional biological TEM sections,^52^, ^53^ it is thin enough for electron transparency and to resist dissociation in water.

For Methods D and E, which rely exclusively on OsO_4_ vapour fixation without aldehyde cross‐linking, OsO_4_ penetration into the bulk material was limited. Therefore, it was preferable to section from the uppermost few micrometeres of the sample surface, where OsO_4_ cross‐linking is most effective.

Bioplastic specimens prepared via Method E (USP‐140°C‐15 kN and BMSP‐140°C‐7 kN) were cut at 100–130 nm. Samples prepared using Method E2 and BMSP‐140°C‐7 kN (48 h OsO_4_ vapour exposure), sectioned at lower thicknesses, exhibited noticeably improved cohesion and cutting stability compared to samples prepared using E1 (24 h exposure), suggesting that longer vapour fixation enhances bioplastic matrix cohesion in E2 protocol. Once uniform gold–silver colouration and consistent section thickness were confirmed, all sections were collected onto copper TEM grids for imaging.

### 2.6 TEM imaging

Sectioned Spirulina samples mounted on copper TEM grids were imaged with a JEOL 1400 (JEOL USA Inc.) transmission electron microscope equipped with a LaB_6_ filament. Samples embedded in Spurr's resin (Methods A–C and E) were imaged at 120 kV, while those embedded in Lowicryl K4M (Method D) were imaged at 80 kV. The images were acquired with an AMT XR11 CCD camera (AMT).

## 3 RESULTS AND DISCUSSION

**FIGURE 2:**
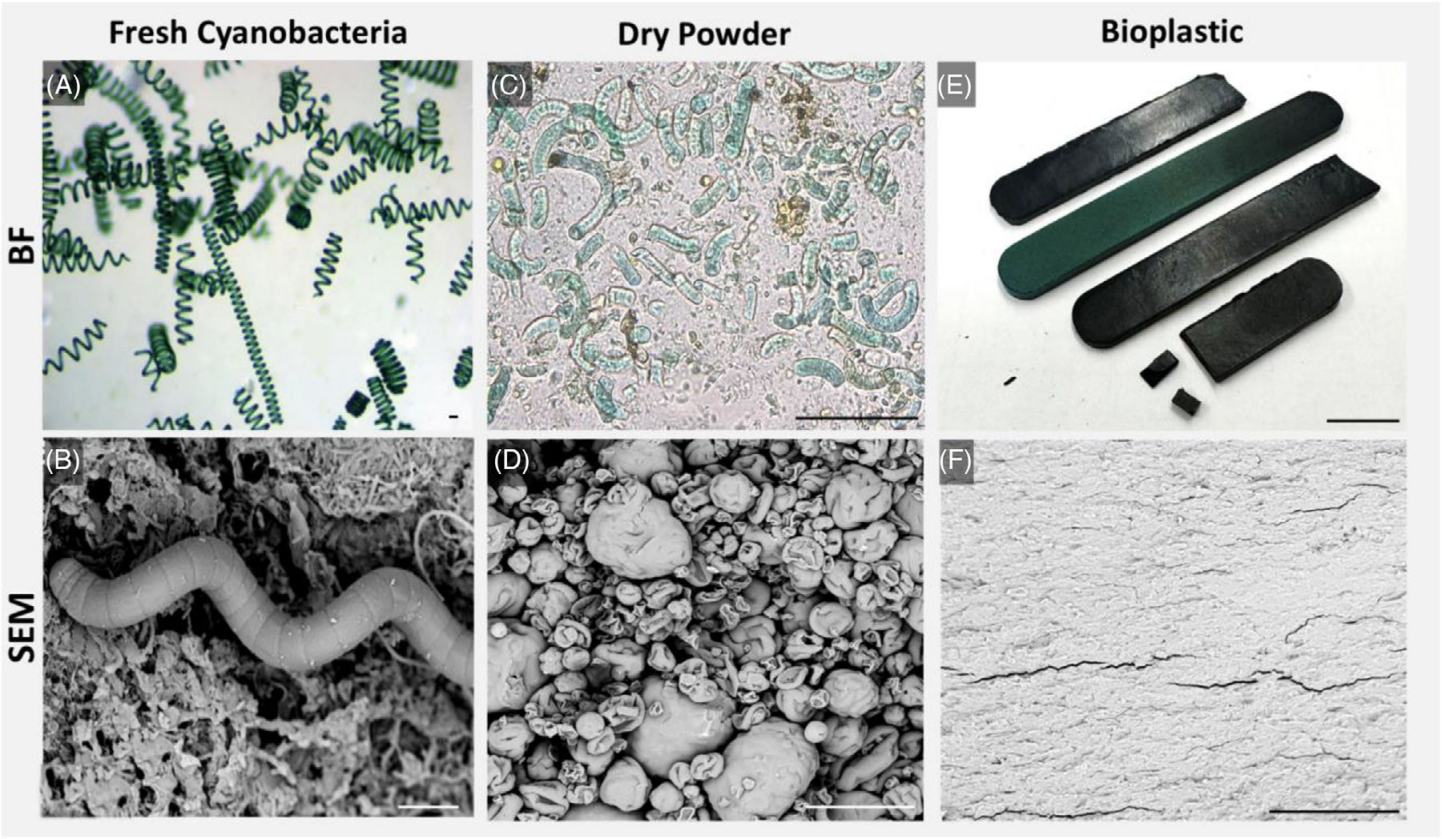
Morphological transformation of Spirulina from fresh cyanobacteria to bioplastic. Fresh Spirulina trichomes imaged by bright‐field (BF) microscopy (A) and scanning electron microscopy (SEM) (B). Purchased dried Spirulina powder observed by BF microscopy (C) and SEM (D). Fabricated Spirulina bioplastic (hot‐pressed at 150°C‐15 kN for 10.5 min from USP‐powder) shown as a macroscopic BF photograph (E) and corresponding SEM surface morphology (F). Scale bars for (A), (B), (D and F), (C), and (E) are 20 µm, 10 µm, 80 µm, 100 µm, and 10 mm, respectively. Images (A) and (B) were adopted from Sili et al.^15^ and Nowicka‐Krawczyk et al.,^11^ respectively.

In order to evaluate the suitability of the different TEM sample preparation techniques developed here, we first analysed the anatomy and structure of Spirulina under different conditions (USP powder and fresh state) and at different length scales using bright‐field (BF) and scanning electron microscopy (SEM). Figure 2 presents images of Spirulina cyanobacteria in the fresh state (Figure 2A and B), dried USP powder (Figure 2C and D), and Spirulina bioplastic (Figure 2E and F).

Spirulina consists of a multicellular, helical trichome structure (50–500 µm in length and 3–12 µm in diameter), in which individual cells divide by binary fission (Figure 2A and B).^10^, ^15^, ^54^ The dried Spirulina used for bioplastic fabrication exhibits a distinct morphology compared to fresh cyanobacteria. Under BF microscopy, the USP powder contains a heterogeneous mixture of impurities, disrupted helical trichomes, intact cells, as well as partially fragmented cells (Figure 2C).

Correspondingly, SEM imaging reveals a predominantly globular morphology (agglomerated Spirulina trichomes and impurities) that contrasts with the intact structure of fresh cyanobacteria (Figure 2D). Once the powder is processed into a bioplastic (Figure 2E), the cyanobacteria appear fused together, forming a continuous, coherent matrix visible in SEM micrographs (Figure 2F). However, cell‐cell interactions, bonding, and ultrastructural transformation of the bioplastic are not observable in this scale. It's therefore essential to develop a new TEM preparation method for observing the morphology of Spirulina bioplastic.

### 3.1 Method A

**FIGURE 3:**
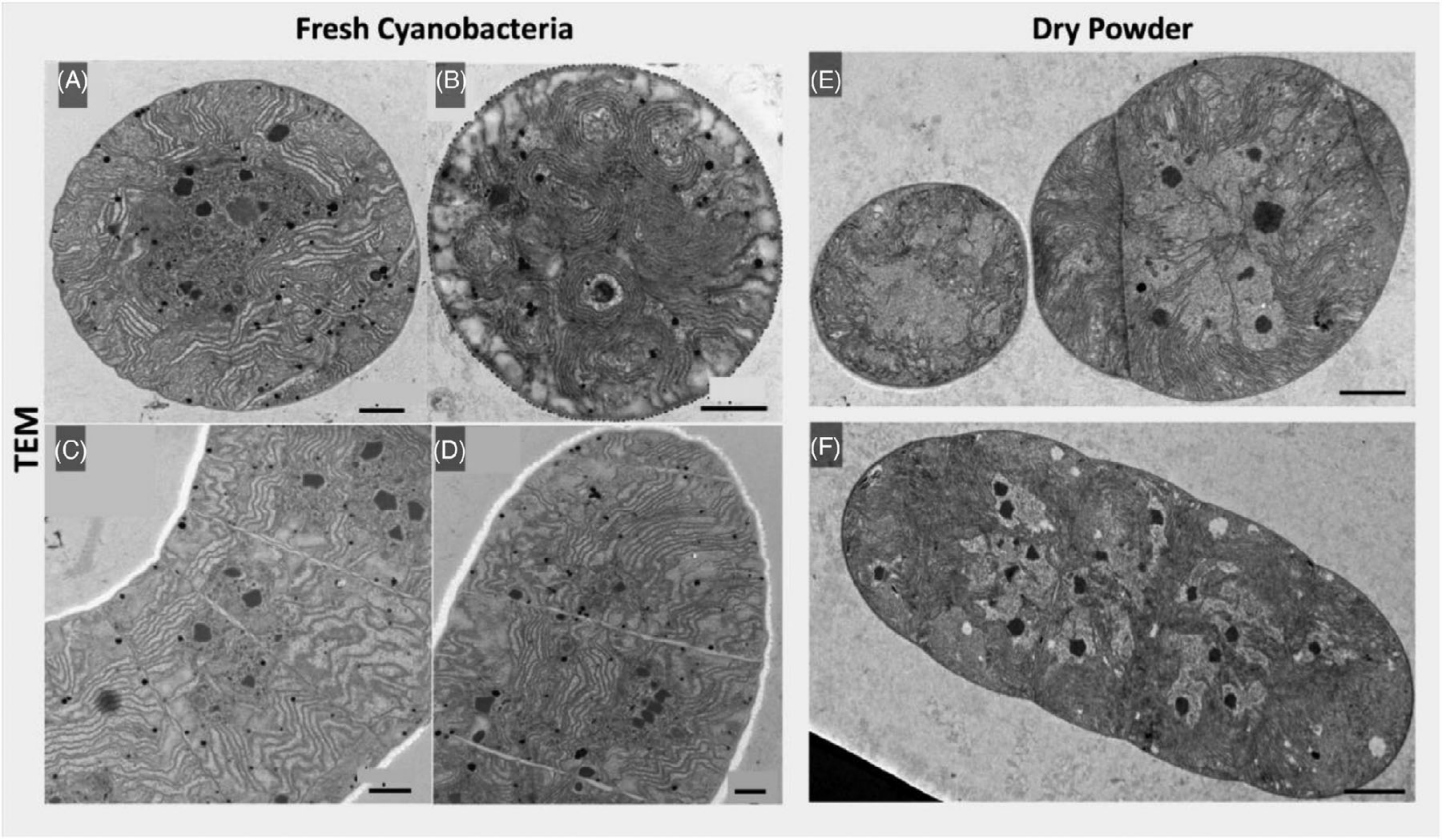
TEM images of transverse (A, B), longitudinal (C, D, and F), and oblique (E) sections of Spirulina cells. (A–D) Micrographs of cyanobacteria prepared for TEM in the fresh state were adopted from Nowicka‐Krawczyk et al.^11^ (E, F) Images of dried Spirulina powder (USP powder) prepared via Method A. Scale bars for (A–D), and (E and F) are 1 and 2 µm, respectively.

**FIGURE 4:**
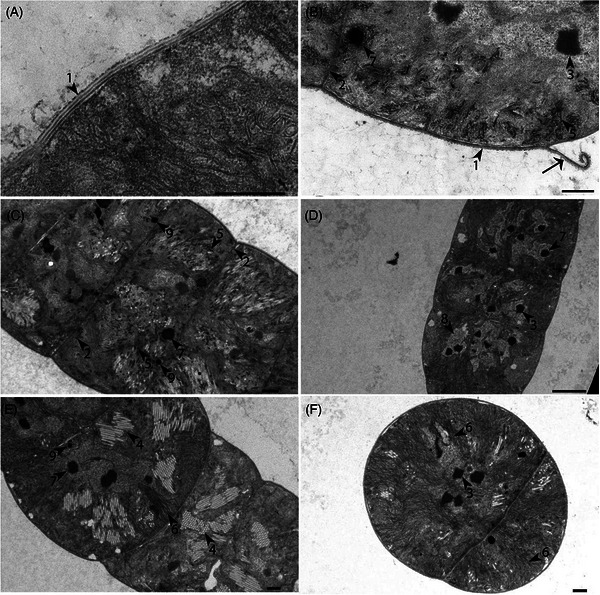
High‐magnification TEM images of detailed views of Spirulina cells prepared using Method A. (A and F) Transverse and (B–E) longitudinal sections of intact cells. Arrowheads indicate: (1) Spirulina cell wall, (2) septum, (3) carboxysome, (4) gas vesicles, (5) polyglucan granule, (6) thylakoid, (7) cyanophycin, (8) ribosome‐region, and (9) lipid droplets. The arrow in (B) indicates a detached cell wall. The scale bar in (B) is 2 µm, and those in (A, C–F) are 500 nm.

USP powder was first examined prior to thermomechanical processing to establish a baseline of the native cellular morphology using TEM (Figures 3E and F and 4). For clarity, these images show intact cells from the as‐received powder, which comprises a mixed population of intact and partially fragmented cells, trichomes, and impurities. Notably, TEM images of dried Spirulina powder prepared using Method A (Figure 3E and F) are very similar to those collected for fresh Spirulina reported by Nowicka‐Krawczyk et al. (Figure 3A–D).^11^

Compared to fresh cyanobacteria images (Figure 3A–D), the shape and dimensions of the cells in USP powder (Figure 3E and F) (3–12 µm in diameter and 2–5 µm in length, depending on the orientation [cross‐sectional or longitudinal]^9^, ^14^, ^20^, ^54^) appear unchanged and most of the cellular components can be observed in higher magnification TEM images (Figure 4). For convenience, Table 2 itemises the Spirulina cell components (Labels 1–9) and artefacts and features (Labels 10–15) observed for each tested protocol. What follows is a description of the cellular features of Spirulina observed using Method A (Figure 4A–F).

**TABLE 2:** List of used abbreviations related to cell components, fixation artefacts, and other features for TEM images.

Spirulina cell components						Artefacts and features			
1	Cell wall	4	Gas vesicle	7	Cyanophycin	10	Resin island	13	Oval‐shaped structure
2	Septum	5	Polyglucan	8	Ribosome region	11	Holes	14	Contrasted cell wall
3	Carboxysome	6	Thylakoid	9	Lipid droplet	12	Swirling structure	15	Impurities

The Spirulina cell wall is composed of four distinct layers (L‐I through L‐IV) (Label 1; Figure 4A and B), approximately 60 nm thick in total.^54^, ^55^, ^56^, ^57^, ^58^ The innermost layer, L‐I, is fibrillar, primarily composed of β‐1,2‐glucan microfibrils. Adjacent to this is the L‐II layer, made up of peptidoglycan (murein), forming a three‐dimensional sugar‐peptide matrix.^18^, ^56^, ^58^ Both L‐I and L‐II contribute to the cyanobacteria cell wall's structural integrity.^55^, ^56^, ^58^ The L‐III layer comprises loosely arranged fibrils with the same composition as L‐I. The L‐IV is the outermost membrane layer. This layer is covered by a protective sheath that typically contains acidic polysaccharides, lipopolysaccharides, vitamins, and minerals, enriching the cell surface composition.^56^, ^57^, ^59^, ^60^ In addition, adjacent Spirulina cells within trichomes are separated by an inner wall septum (Label 2; Figure 4C) with 30–45 nm thickness visible in TEM, composed of a three‐layered structure in which an L‐II layer is enclosed between two L‐I layers.^55^, ^57^ The individual layers of the septum cannot be distinguished in the micrographs presented here.

The other components we can observe in dried Spirulina powder include carboxysomes (Label 3; Figure 4B,D, and E), which have diameters up to 500 nm and are also known as polyhedral bodies. Carboxysomes are proteinaceous cellular structures with sharp edges that contain the ribulose‐1,5‐bisphosphate carboxylase enzyme.^16^, ^54^, ^57^ The gas vacuoles in cyanobacteria (as large as 1500 nm) (Label 4; Figure 4E) contain stacks of rod—and honeycomb‐shaped gas vesicle substructures. The gas vesicles (hollow) are held together through layers composed of various proteins.^57^, ^61^ Polyglucan granules (10–30 nm), or α‐granules (Label 5; Figure 4B and C), are small circular and rod‐shaped components composed of highly branched glucose polymers located between thylakoid bundles.^9^, ^57^

Thylakoids (Label 6; Figure 4E and F) (10–30 nm thick) are photosynthetic lamellae containing chlorophyll and carotenoids complexed with protein.^14^, ^15^, ^57^, ^58^ Phycobilisomes with 10–20 nm diameter, attached to the thylakoids, are protein complexes responsible for the characteristic blue‐green colour of Spirulina. In Spirulina, the dominant phycobiliproteins are C‐phycocyanin and allophycocyanin, together making up about 20% of the cell's dry weight, with C‐phycocyanin being the most abundant protein‐pigment complex.^62^, ^63^, ^64^ Proteinaceous cyanophycin (Label 7; Figure 4B–E), with diameters as large as 1000 nm, can also be observed. These spherical components are storage biopolymers composed of arginine and aspartic acid residues.^9^, ^54^, ^57^ Ribosome‐rich regions (Label 8; Figure 4D), consisting of rRNA, protein, and fibrils of DNA, are located in the thylakoid‐free regions.^17^, ^35^ Small lipid droplets (Label 9; Figure 4C and E) or lipid bodies (100–200 nm) are another component scattered among the thylakoid bundle or thylakoid‐free areas.^9^, ^54^, ^65^

The rest of the cellular structures of Spirulina, including the mesosome and cylindrical bodies, are absent or are not detectable in TEM.^9^, ^14^, ^15^, ^54^ Based on Eykelenburg's study on Spirulina ultrastructure, the mesosome (300–400 nm) is a membrane‐like structure produced from the inner folding of the cytoplasmic membrane ^9^, ^57^, ^66^ and cylindrical bodies (100–200 nm) are cylindrical lamellar membranes located in the nucleoplasmic regions.^3^, ^54^

In addition to unidentified cellular components, we observed minor morphological differences between fresh and dried Spirulina in TEM. In Figure 4B, the arrow indicates a curled cell wall, likely due to the fragile state of the cell wall resulting from dehydration of Spirulina biomass and subsequent infiltration steps. In the subsequent images of Spirulina bioplastic, various swirling structures are also observed (Label 12). Although not every cellular structure could be identified, the implementation of Method A demonstrated that dried Spirulina powder could be successfully imaged using TEM.

The following techniques (B–E) focus on imaging Spirulina bioplastics: (1) USP‐140°C‐15 kN, samples prepared from as‐received USP powder and studied via Methods B–E. (2) BMSP‐140°C‐7 kN, a cell‐disrupted bioplastic sample prepared via Method E1. These bioplastics exhibit microstructures that differ markedly from those of dried Spirulina powder. Each image set is analysed to assess how the respective TEM preparation method influences structural preservation and artefact formation relative to the initial morphology.

### 3.2 Method B

**FIGURE 5:**
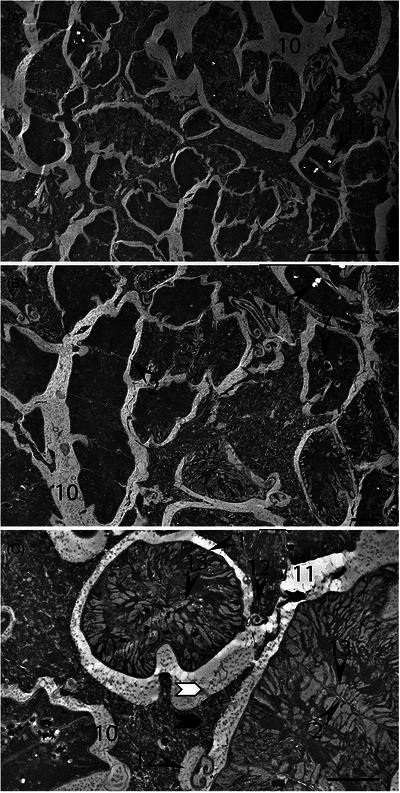
TEM images of USP‐140°C‐15 kN prepared for TEM using Method B (A–C). Black and white arrows point out the cell fragment and cytoplasmic components, respectively. Arrowheads indicate (1) cell wall, (2) septum, (7) cyanophycin, (10) resin island, (11) holes, (12) swirling structures, and (13) oval‐shaped structure. The scale bar in (A) is 10 µm, and the scale bars in (B) and (C) are 2 µm.

Figure 5 displays TEM images of Spirulina bioplastic prepared using Method B, which followed a very similar preparation protocol to Method A. In Figure 5A–C, resin ‘islands’ (Label 10) appear both at cell interfaces and within cytoplasmic regions as a consequence of liquid‐phase infiltration. Within those regions, several immobilised intracellular constituents (white and black arrows; Figure 5C) are detectable outside the cell boundaries. These compartments, including a cell fragment (black arrow) and cytoplasmic components (white arrow), are visibly suspended within the resin islands. Neither of these features are present in the micrographs of USP powder (Figures 3E and F and 4), suggesting that the primary fixative step solubilises and mobilises cytoplasmic components of bioplastics. During subsequent resin infiltration and polymerisation, these displaced constituents become immobilised within the embedding medium.

In Figure 5C, intracellular constituents (white and black arrows) within the resin islands are likely to arise from the replacement of the bioplastic's internal bonding with aldehyde–Spirulina interactions. During TEM preparation, aldehyde fixatives react with proteins, forming covalent crosslinks via amino acid side chains.^13^, ^22^ In Spirulina bioplastics, cohesion between neighbouring cells likely arises from hydrogen bonding and fibrillar interlocking of biopolymers in neighbouring cell walls, as well as aggregated proteins from disrupted cells filling the spaces between them.^3^, ^67^ Aldehyde penetration can interfere with these intercellular protein–protein interactions, thereby redirecting reactive groups toward fixative binding rather than Spirulina protein self‐association.^68^ This disruption of the protein‐rich matrix and cell wall contacts caused by chemical treatments likely contributes to the cell–cell separations and cohesion loss observed as resin island artefacts (Label 10; Figure 5A–C) in Spirulina bioplastics prepared by Method B.

Holes (Label 11; Figure 5A–C) are also visible in the bioplastic images, likely resulting from non‐uniform resin infiltration into the processed bioplastic, which creates section discontinuities. At higher magnification (Figure 5C), these discontinuities manifest as localised ruptures within the electron‐transparent sections. In addition to resin islands and holes, there are a number of swirling structures, probably ruptured cell walls (Label 12; Figure 5A and C), visible in the bioplastic specimens prepared using Method B. Cell wall rupture can occur either during processing or during infiltration steps.

The presence or absence of resin islands and holes serves as baseline criteria for evaluating the successful preservation of the Spirulina bioplastic's initial morphology. Limiting the introduction of artefacts during sample preparation and imaging is a primary objective when studying the morphology of processed materials, as it enables reliable assessment of how variations in processing conditions influence the specimen's structure. Based on these criteria, the widespread occurrence of both resin islands and holes in Figure 5 indicates that Method B is inappropriate for preparing Spirulina bioplastic samples. Accordingly, we explored different TEM preparation techniques—such as heat‐induced Lowicryl polymerisation and modified infiltration protocols—to minimise these artefacts throughout this study.

**FIGURE 6:**
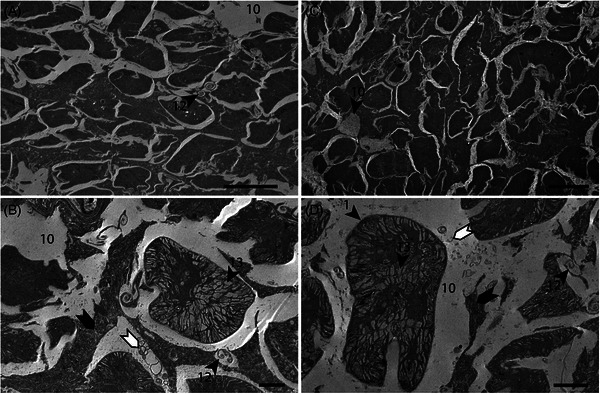
TEM images of USP‐140°C‐15 kN prepared using methods C1 (A, B) and C2 (C, D). Black and white arrows point out the cell fragment and cytoplasmic components, respectively. Arrowheads indicate: (1) cell wall, (10) resin island, (12) swirling structures, and (13) oval‐shaped structure. The scale bars for (A and C) are 10 µm and for (B and D) are 2 µm.

**FIGURE 7:**
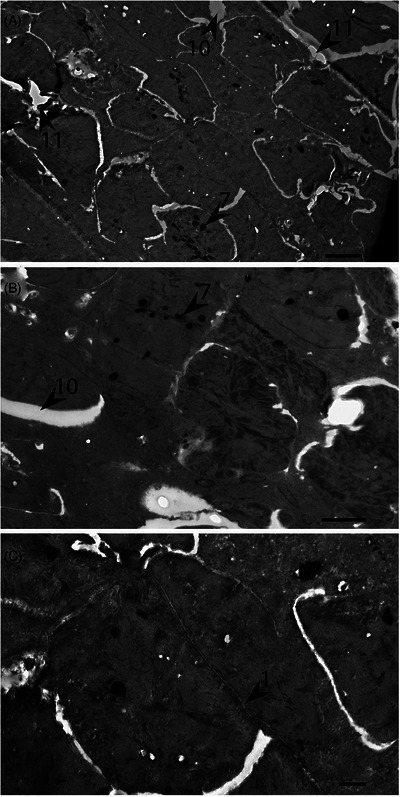
TEM images of USP‐140°C‐15 kN sample prepared using Method D (A–C). The arrowheads show: (1) cell wall, (7) cyanophycin granule, (10) resin island, and (11) holes. The scale bars in (A and B) are 2 µm, and the scale bar in (C) is 500 nm.

**FIGURE 8:**
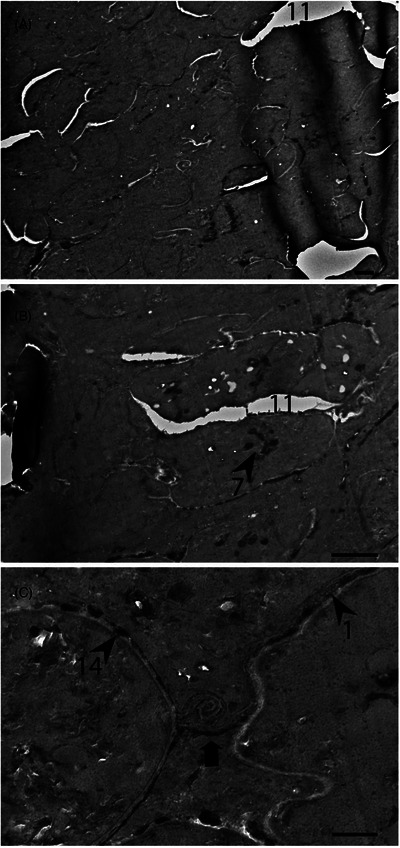
TEM images of sample USP‐140°C‐15 kN prepared via Method E1 (A, B). Labels indicate (1) cell wall, (7) cyanophycin granule, (10) holes, (14) contrasted cell wall, and (a black arrow) cell wall or cross‐wall interaction. Scale bars for (A and B) are 2 µm and (C) is 500 nm.

**FIGURE 9:**
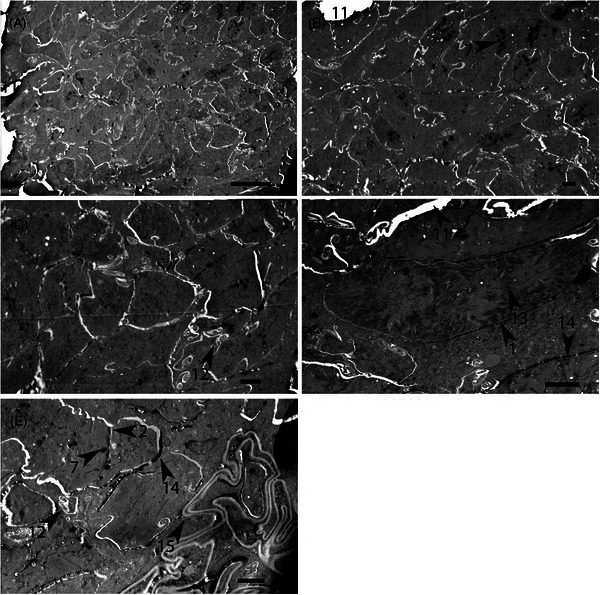
TEM images of USP‐140°C‐15 kN bioplastic samples prepared by Method E2 (A–E) were exposed to 48 h of OsO_4_ vapour exposure. Label indicates (1) cell wall, (2) septum, (7) cyanophycin granule, (11) holes, (12) swirling structure, (13) oval‐shaped structures, (14) contrasted cell wall, and (15) impurities. Scale bars for (A) are 10 µm and (B–E) are 2 µm.

**FIGURE 10:**
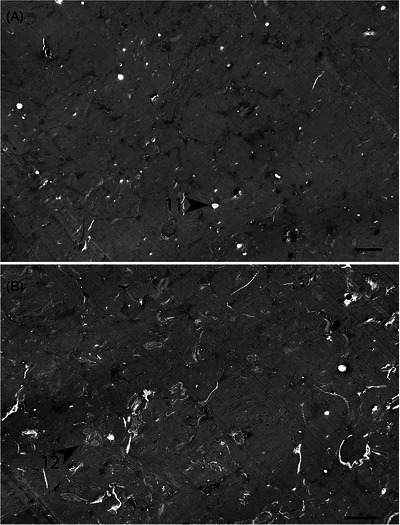
TEM images of the BMSP‐140°C‐7 kN Spirulina bioplastic prepared via Method E1; (11) holes and (12) swirling structures. Scale bars are 2 µm.

It is also interesting to note that, compared to the dried powder prepared using Method A, the Spirulina bioplastic prepared using Method B shows less structural detail within the cells. The only cellular components visible are the cell wall (Label 1 Figure 5C), septum (Label 2; Figure 5B and C) and the cyanophycin and or carboxysome (Label 7 or 3; Figure 5B). Due to their similar size and shape, cyanophycin (Label 7) and carboxysome (Label 3) will be referenced only using Label 7 in Figures 5, 6, 7, 8, 9, 10. Another unique feature developed after bioplastic conversion is the formation of oval‐shaped structures (Label 13; Figure 5C) derived from cytoplasmic components. These ovular features were not observed in images of the dried Spirulina (Figure 4). Compared with intact Spirulina cells in Figures 3 and 4, the changes in cellular features in the bioplastic micrographs could be explained by sample incompatibility with Method B or by molecular rearrangements induced by elevated temperature and pressure during bioplastic hot‐pressing.

### 3.3 Method C

Method C eliminated the first fixative step (described in Method B) to reduce effects induced by aldehyde fixation on cell–cell cohesion. Instead, direct liquid‐phase OsO_4_ fixation with ethanol dehydration, followed by either acetone (C1) or propylene oxide (C2) rinses (Figure 6A–D), was utilised for this protocol. Both Method C1 (Figure 6A and B) and Method C2 (Figure 6C and D) produce micrographs that exhibit continuous sections without macroscopic holes; however, for both C1 (Figure 6B) and C2 (Figure 6D), there are pronounced resin‐filled gaps (Label 10). In addition, as in Method B, suspended‐immobilised intracellular constituents, including cell fragments and cytoplasmic components (black and white arrows, respectively; Figure 6B and D), are still visible within resin islands between adjacent cells.

Although omitting the aldehyde fixation in Method C was intended to mitigate fixative‐induced cell–cell separation, morphological disruption persists due to interactions between infiltration reagents and protein‐based components. In Method B, this effect is primarily associated with aldehyde fixatives, whereas in Method C, OsO_4_ fixation followed by ethanol and acetone, or propylene oxide dehydration, produces comparable artefacts. The presence of resin islands (Label 10; Figure 6A–D) and isolated intercellular constituents (black and white arrows) is attributed to the direct exposure of the processed biomaterial to liquid‐phase dehydrating and contrasting agents, which can interact with and extract protein‐ and lipid‐rich components, thereby facilitating resin infiltration into the intracellular matrix.^37^, ^69^, ^70^

For samples prepared using Method C, Spirulina cell wall (Label 1; Figure 6D) is the only native cellular component that can be identified. Other visible features developed during bioplastic fabrication include swirling structures (Label 12; Figure 6A,B, and D) and oval‐shaped features (Label 13; Figure 6A and D) observed in both C1 and C2. Finally, we note that changing the second solvent used during sample dehydration (acetone vs. propylene oxide) does not affect the morphology observed during imaging (C1 vs. C2).

### 3.4 Method D

This method was developed to minimise direct contact between Spirulina bioplastics and liquid contrasting reagents through OsO_4_ vapour fixation. Additionally, Method D was designed to improve resin infiltration by using the lower‐viscosity Lowicryl K4M compared to Spurr's resin. Overall, the approach stabilises structural components via vapour‐phase crosslinking, avoids the disruptive effects of conventional infiltration (Method B), and reduces artefacts associated with liquid‐phase contrasting and dehydration (Methods C1 and C2).

Figure 7 presents a bioplastic specimen prepared for TEM using Method D. By avoiding the application of liquids to the bioplastic surface, Method D produced micrographs with significantly fewer infiltration‐related artefacts. However, holes (Label 11; Figure 7A) and resin islands (Label 10; Figure 7A and B) remain, and image contrast and resolution were reduced, disrupting the overall morphology. The persistence of holes within the TEM sections imaged using Method D may be associated with the hydrophilicity of Lowicryl K4M. Exposure to water during sectioning compromised the consistency of the Lowicryl matrix, making it difficult to obtain ultrathin sections.^53^

While some cellular features, such as the cell wall (Label 1; Figure 7C) and cyanophycin granules (Label 7; Figure 7A and B) are visible, the sectioning difficulty, undesirable contrast, and the continued presence of artefacts indicate incomplete preservation of the Spirulina bioplastic's structural integrity and continued disruption of cell–cell interactions.

### 3.5 Method E

Method E incorporates the same vapour fixation technique as Method D but uses Spurr's resin instead of Lowicryl K4M. Neither aldehyde fixatives nor liquid dehydrants are included in Method E. To study the impact of vapour exposure time, samples were either exposed to a 2% OsO_4_ deposition for 24 h (Method E1), or for 48 h (Method E2). Compared with the previous methods discussed, OsO4 vapour fixation without aldehyde fixation or dehydration yielded the most faithful visualisation of Spirulina bioplastic morphology and ultrastructural transformation, without introducing numerous TEM artefacts.

While resin islands were not observed in samples prepared using Method E1, large holes were present (Label 11; Figure 8A and B). The visible holes are larger than those observed after sample preparation using Methods B–D, suggesting that a 24‐h exposure is insufficient to maintain the structural integrity of the thin‐sectioned bioplastic. Similar to samples prepared using Method D, ultramicrotomy of samples prepared using Method E1 was more challenging, as the sections disintegrated rapidly in water, necessitating relatively thick sections of ∼130 nm. However, despite the presence of holes, Method E1 did enable the observation of a well‐contrasted cell wall bonded to the surrounding matrix (Label 14; Figure 8C). In addition, cell walls (Label 1; Figure 8C) from two neighbouring cells were observed in close contact (Figure 8C, arrow), representing a level of structural preservation not achieved with the previous preparation methods. The successful detection of these two features suggests that osmium tetroxide vapour can act as an effective cross‐linker, helping to preserve bioplastic integrity without inducing detachment between adjacent cell walls or between the cell wall and the intracellular matrix.

For Method E2 (Figure 9), we modified Method E1 and increased the OsO_4_ vapor exposure time to 48 h. Method E2 combines the benefits of enhanced fixation with improved section integrity, allowing for more reliable ultramicrotomy and the preparation of thinner sections (∼100 nm). While a 24‐h exposure was sufficient to reveal some subfeatures, like cell–cell wall contacts (Figure 8C, arrow), it was insufficient to produce completely faithful observations of the bioplastic microstructure.

Method E2 showed markedly improved image contrast and structural preservation. Cell wall–cell wall contacts were clearly resolved, and intracellular subfeatures—including cell walls (Label 1; Figure 9D), septums (Label 2; Figure 9E), cyanophycin granules (Label 7; Figure 9E)—as well as processing‐induced oval‐shaped structures (Label 13; Figure 9D), were well preserved. The swirling features (Label 12; Figure 9C) previously noted were also observed. The relatively uniform matrix surrounding the ruptured cell walls suggests that they are likely intrinsic to the bioplastic formation process, however, infiltration techniques (Methods B and C) can cause further damage to cell walls during sample preparation and increase the prevalence of feature 12 in the resulting images.

Figure 9 shows that some holes (Label 11; Figure 9A,B, and D) were still present within and around the edge of the matrix in samples produced using Method E2. However, comparison of Figures 8 and 9 indicates that voids are also inherently present between cells due to weak bonding between cell walls and the intracellular matrix. Given the overall matrix integrity, these features are more likely processing‐induced defects between the cells (labelled 11; Figure 9D) that become slightly larger during infiltration processing.

The presence of well‐contrasted cell walls on both high‐ (Figure 9B–E) and low‐magnification (Figure 9A) images provides further support for our hypothesis that OsO_4_ acts as a binder, strengthening lipid–protein interactions by cross‐linking them and preserving matrix integrity—an outcome not achieved with Method E1.^22^, ^34^, ^35^ The micrographs produced using Method E2 (Figure 9) indicate that extended OsO_4_ vapour exposure (48 h) is particularly advantageous for retaining bioplastics' cellular morphology and best meets our goals of reducing artefacts (in this case, often resin infiltration between cells) and providing superior image quality that reveals the substructure.

Previous works have shown that, in addition to processing conditions, Spirulina powder preprocessing can affect bioplastic structure and performance.^3^ As shown in Figure 1F, Iyer et al. demonstrate that disrupting Spirulina cells via ultrasonication results in bioplastics (SSP‐140°C‐35 kN) with higher flexural strength than Spirulina bioplastics prepared using USP powder (USP‐140°C‐35 kN).^3^ To ensure that our TEM preparation techniques are resilient to changes in bioplastic processing, we explored ball milling as an alternative cell‐disruption technique for Spirulina^71^, ^72^ and investigated the resulting morphological changes.

Alongside USP‐140°C‐15 kN bioplastic, a cell‐disrupted bioplastic specimen (BMSP‐140°C‐7 kN) was also prepared using Method E1. Compared to images of the USP bioplastics obtained via Method E (Figures 8 and 9), micrographs of BMSP‐140°C‐7 kN (Figure 10) demonstrate uniform morphology consisting of a mixture of cyanobacterial intracellular components. We hypothesise that ball‐milling fractures trichomes, intact and fragmented cells, cell walls, and protoplasts, thereby reducing the number of cell wall–cell wall interfaces and leading to a more cohesive matrix.

Despite the lower processing force (7 kN vs. 15 kN) and shorter OsO_4_ vapour exposure compared to Method E2, the preparation of BMSP‐140°C‐7 kN using E1 enables the observation of the homogeneous matrix, showing a limited number of holes (Label 11; Figure 10A) along with swirled cell‐wall fragments (Label 12; Figure 10B) and several well‐contrasted intracellular components. This increased uniformity, induced by cell disruption prior to the thermomechanical transformation, likely improves overall sample cohesion during TEM preparation, facilitates microtomy, and indicates that 24 h of OsO_4_ vapour exposure was sufficient to preserve the structural integrity of this specimen.

### 3.6 Methods comparison

**FIGURE 11:**
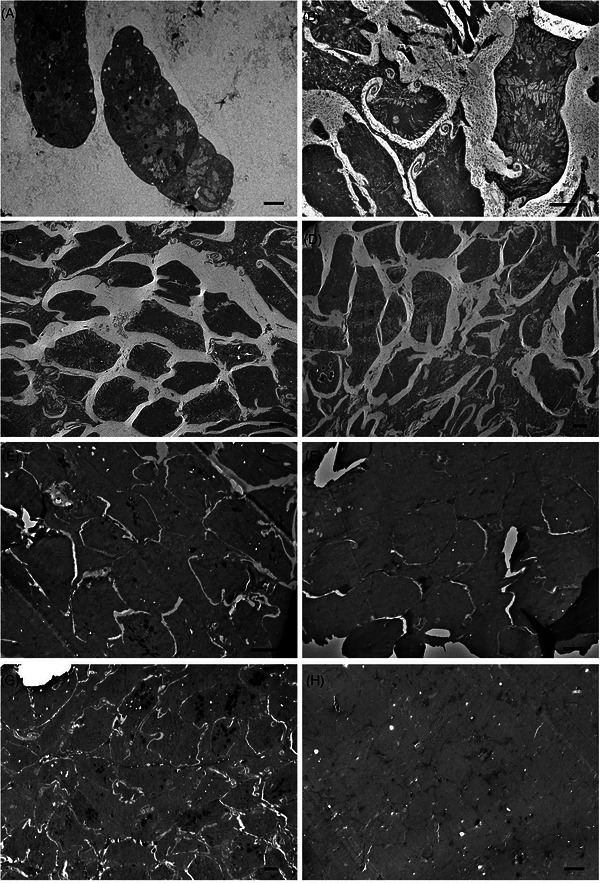
Dried Spirulina and bioplastic TEM images prepared through Methods A–E. Method A (A) is used for USP powder. Methods B (B), C1 (C), C2 (D), D (E), E1 (F), and E2 (G) were employed on USP‐140°C‐15 kN bioplastic. BMSP‐140°C‐7 kN processed from BMSP powder and was prepared via E1 (H). All scale bars are 2 µm.

Figure 11 summarises the TEM preparation methods evaluated in this study: Method A applied to USP powder (Figure 11A) and Methods B–E applied to USP‐140°C‐15 kN bioplastics made from USP powder (Figure 11B–G). To assess the applicability of the vapour fixation protocol to Spirulina bioplastics with different morphologies, cell‐disrupted BMSP‐140°C‐7 kN was also tested using Method E1 (Figure 11H).

Method A was selected based on its successful application to imaging fresh Spirulina cyanobacteria.^11^, ^14^ This method is suitable for preserving and imaging dried powder, including intact and fragmented cells (Figure 11A); however, applying the same aldehyde‐based infiltration protocol to thermomechanically transformed Spirulina bioplastics (Method B) proved unsuitable (Figure 11B). In this case, aldehyde fixation disrupted bioplastic chemical bonding and promoted resin penetration between cells, leading to significant infiltration‐induced artefacts. In Method C, the primary aldehyde fixative was eliminated, and OsO_4_ fixation was combined with alternative dehydration agents. However, this modification did not resolve the issue, as infiltration‐related artefacts persisted in both C1 (acetone; Figure 11C) and C2 (propylene oxide; Figure 11D).

Method D excluded both aldehyde fixation and dehydration using organic solvents. Instead, this method employed 24 h OsO_4_ vapour exposure (vapour fixation technique) followed by embedding in low‐viscosity hydrophilic Lowicryl K4M resin, to improve resin penetration (Figure 11E). Although this approach reduced the size and number of the resin island artefacts compared with Methods B and C, morphological disruption of the bioplastic matrix remained evident.

Method E1 adapted Method D, applying 24 h OsO_4_ vapour exposure followed by hydrophobic resin embedding (Spurr's), rather than Lowicryl K4M (Figure 11F). While resin islands were no longer observed—suggesting that osmium enhanced matrix cohesion—large holes between cells remained, limiting the ability to observe a coherent bioplastic matrix. To address this limitation, Method E2 extended the OsO_4_ vapour exposure to 48 h prior to resin embedding (Figure 11G). This approach reduced the size and quantity of holes while simultaneously preserving processing‐induced ultrastructural transformations in Spirulina bioplastics—an outcome not achieved using Methods B–D.

An effective TEM preparation protocol for this emerging class of biomaterials should produce high‐quality micrographs across varying processing conditions. Protocol E1 was applied to cell‐disrupted Spirulina bioplastics (BMSP‐140°C‐7 kN). This sample was prepared via BMSP powder under different processing forces (kN). The morphology observed in Figure 11H differs from USP‐140°C‐15 kN bioplastic, which exhibits a more uniform structure without gaps between adjacent cell walls.

The preservation of structural features in the ball‐milled bioplastic shows that Method E effectively captures morphological differences, indicating its broad applicability to Spirulina‐based bioplastics. The vapour fixation technique, followed by Spurr's embedding, reduces preparation artefacts while maintaining structural integrity, enabling reliable nano‐ to micro‐scale analysis and making it an effective TEM prep for Spirulina bioplastics.

## 4 SUMMARY

To investigate the ultrastructural transformation of dried cyanobacteria to cohesive Spirulina bioplastics using thermomechanical processing, we developed tailored TEM imaging protocols. Custom preparatory methods were designed to significantly reduce preparation‐induced artefacts, such as holes and resin impregnation, compared with previously reported approaches. By using custom 2% OsO_4_ vapour fixation, different exposure times, and hydrophobic resin (Spurr's) embedding, we successfully obtained the first high‐fidelity TEM images of this new material class after various processing conditions.
